# Biomimetic nanovesicle co-delivery system impairs energy metabolism for cancer treatment

**DOI:** 10.1186/s12951-023-02061-4

**Published:** 2023-08-26

**Authors:** Yongmei Zhao, Yan Zhu, Kai Ding, Shanshan Li, Tianqing Liu

**Affiliations:** 1 School of Pharmacy, Nantong University, Nantong, China; 2 School of Pharmacy, Bengbu Medical College, Bengbu, China; 3 NICM Health Research Institute, Western Sydney University, Westmead, NSW 2145 Australia

**Keywords:** Biomimetic nanomedicines, Energy metabolism, M1 macrophage, Metformin, 3-Bromopyruvate, Cancer therapy

## Abstract

Metabolic reprogramming in cancer cells plays a crucial role in cancer development, metastasis and invasion. Cancer cells have a unique metabolism profile that could switch between glycolysis and oxidative phosphorylation (OXPHOS) in order to satisfy a higher proliferative rate and enable survival in tumor microenvironment. Although dietary-based cancer starvation therapy has shown some positive outcomes for cancer treatment, it is difficult for patients to persist for a long time due to the adverse effects. Here in this study, we developed a specific M1 macrophage-derived membrane-based drug delivery system for breast cancer treatment. Both metformin and 3-Bromopyruvate were loaded into the engineered cell membrane-based biomimetic carriers (Met-3BP-Lip@M1) for the shutdown of energy metabolism in cancer cells via simultaneous inhibition of both glycolysis and oxygen consumption. The in vitro studies showed that Met-3BP-Lip@M1 had excellent cancer cell uptake and enhanced cancer cell apoptosis via cell cycle arrest. Our results also demonstrated that this novel biomimetic nanomedicine-based cancer starvation therapy synergistically improved the therapeutic efficiency against breast cancer cells by blocking energy metabolic pathways, which resulted in a significant reduction of cancer cell proliferation, 3D tumor spheroid growth as well as in vivo tumor growth.

## Introduction

Cancer is the leading cause of death. According to the World Cancer Report 2020, there were around 19.3 million new cancer cases and almost 10.0 million cancer deaths in 2020 throughout the world [1]. Although extensive efforts have been made to treat cancer, conventional methods, such as surgery, chemotherapy, and radiotherapy, achieve unsatisfactory therapeutic outcomes due to low targeting ability, side effects, and the possibility of recurrence and metastases. Metabolic reprogramming in cancer cells plays a crucial role in cancer development, metastasis and invasion. The altered energy metabolism has been well documented in cancer cells and Warburg effect is recognized as a hallmark of tumors. Tumor cells exhibit enhanced glycolytic activity compared with normal cells to satisfy a higher proliferative rate and enable survival in tumor microenvironment [2, 3]. This energy metabolic shift has been used as a new target for anti-cancer therapy [4]. However, the therapeutic efficacy of current therapies associated with cancer cell energy metabolism is limited due to poor drug bioavailability, metabolic plasticity of tumor cells, and complexity of tumor microenvironment. It is necessary to develop efficient treatment strategies to control tumor cell energy metabolism.

Based on the fact that tumor cells can switch between glycolysis and oxidative phosphorylation (OXPHOS) to adapt the metabolic challenges, metabolic reprogramming by targeting these energy metabolism pathways seems to be a viable option. A recent study has demonstrated that cancer starvation therapy via combination of hypoglycemia and metformin can inhibit cancer growth via blocking both glycolytic and OXPHOS activities [5] Metformin (Met) is an insulin resistance drug widely used for type II diabetes treatment [6], and has been used for cancer treatment because of its effects in cell proliferation and metabolism [7, 8]. It can reduce OXPHOS and cellular energy levels via inhibiting mitochondrial complex I [9]. Although the combination of intermittent fasting with metformin treatment has showed some positive effects against cancer, dietary restrictions are frequently accompanied by weight loss, nausea, and immune system impairment, and it is difficult for patients to persist for an extended period of time. Thus, it normally produces unsatisfactory clinical outcomes. Consequently, adopting pharmacological approaches to target metabolic plasticity may be an effective strategy for metabolism-based cancer management. More recently, several co-administration strategies have been developed for simultaneous inhibition of glucose uptake and OXPHOS efficiently reduced tumor growth and metastasis [10, 11]. 3-Bromopyruvate (3BP) is an effective glycolytic inhibitor with excellent anti-cancer efficacy [12, 13]. Although 3BP can significantly induce cancer cell death by disrupting energy metabolism, it has very short half-life. Consequently, there is limited attempt to apply the combination therapy of both Met and 3BP due to variable drug bioavailability and low intratumoral distribution.

Recently, biomimetic nanovesicles have received great attention for drug delivery application due to their long circulation, low immunogenicity, penetration ability through numerous biophysiological barriers [14–18]. Particularly, macrophage membrane-coated nanovesicles which contain macrophage-associated membrane proteins have shown potential as biomimetic delivery systems for tumor targeting without loss of their tumor-homing nature. For example, it has been reported that macrophage membrane-coated liposomes improved delivery to cancer metastatic sites and prolonged the circulation time of nanoparticles in vivo [19, 20]. More interestingly, we have also demonstrated that pro-inflammatory (M1/M1-like) macrophage-derived natural nanovesicles have remarkable drug delivery and immunostimulatory properties [15, 16]. Thus, in this study, liposomes coated with M1 macrophage cell membranes was used as a drug nanocarrier to encapsulate Met and 3BP for simultaneous inhibition of both glycolysis and OXPHOS and shutdown of energy metabolism in cancer cells. Our results showed that this novel cancer starvation therapy based on the M1 macrophage membrane-coated liposomes containing Met and 3BP (Met-3BP-Lip@M1) synergistically improved the therapeutic efficiency against breast cancer cells by blocking energy metabolic pathways which resulted in significant reduction of cancer cell proliferation and 3D tumor spheroid growth, as well as in vivo tumor growth.

## Materials and methods

### Cell culture

Mouse mononuclear macrophage cell line RAW264.7 and mouse breast cancer cell line 4T1 were both purchased from the American Type Culture Collection (ATCC, USA). Both cell lines were grown in the complete DMEM (Dulbecco’s modified Eagle’s medium) medium, supplemented with 10% fetal bovine serum (FBS) and 1% penicillin/streptomycin in a humidified cell culture incubator at 37 °C with 5% CO_2_.

### Preparation of drug-loaded liposomes (Met-3BP-Lip)

Liposomes are synthesized via thin-film hydration by a standard extrusion method. To obtain a liposome membrane, 100 mg of phospholipid and 5 mg of cholesterol were weighed, dissolved in chloroform, evaporated under negative pressure at 37 °C for 4–5 h, and dried in a vacuum oven at 37 °C overnight to completely remove the organic solvent. The required amounts of Met and 3BP were weighed and dissolved in PBS before being added as an aqueous phase to blank liposome membranes to form a homogeneous emulsion. Finally, the above-mentioned liquid was stirred at room temperature for 2 h to be hydrated, and the particle size was controlled by a liposome extruder or an ultrasonic cell crusher. The Met-3BP-Lip emulsion was obtained.

### Extraction of M1 macrophage membranes

M1 macrophage membranes were isolated from the RAW 264.7 cells using previously reported extraction steps with minor modifications to the experimental procedure [15, 19]. Briefly, RAW 264.7 cells were stimulated with 100 ng/mL lipopolysaccharide (LPS, Sigma-Aldrich) for 24 h to induce M1 macrophage polarization [21] Then, M1 macrophages were harvested with a cell scraper at a concentration of 8 × 10^6^ cells/mL and resuspended in 1× Tris-Mg buffer (Beyotime Biotechnology) containing 1 mM phenylmethylsulfonyl fluoride (PMSF). The collected cells were passed through a 400 nm liposome extruder without a polycarbonate membrane and extruded back and forth 20 times to rupture the membrane. The disrupted cell homogenate was mixed with 1 M sucrose to make the final sucrose concentration of 0.25 M. Then, the samples were centrifuged at 2000 rpm for 15 min at 4 °C to collect the supernatant. This process was repeated once more to remove the remaining organelles. Finally, the purified cell membrane pellet was obtained by centrifuging the supernatant at 10,000 rpm for 15 min at 4 °C. The extracted macrophage membranes will be used for preparation of RMEL NPs. The protein content in the purified macrophage cell membrane was determined by a BCA protein concentration assay kit (Beyotime Biotechnology).

### Preparation of Met-3BP-Lip@M1

Met-3BP-Lip@M1 was prepared by a physical extrusion method. Briefly, the purified macrophage membranes were mixed with Met-3BP-Lip@M1 in a volume ratio of 1:1 and then passed through a liposome extruder equipped with a 200 nm polycarbonate membrane for 20 consecutive extruded times.

### Nanoparticle characterizations

The particle size, polydispersity index (PDI), and zeta potential of the nanoparticles were measured using dynamic light scattering (DLS, Malvern Instruments, UK). The morphological features of liposomes were observed by transmission electron microscopy (TEM, JEM-1200EX, Japan). For TEM detection, the samples were diluted with PBS (pH 7.4), added to the copper grid surface and dried before negative staining using uranyl acetate solution (1%, w/v).

### Drug loading and encapsulation efficiency

Drug loading and encapsulation efficiency were determined after ultracentrifugation at 10,000–20,000 rpm for 45 min and methanol-induced demulsification. The amount of Met was detected by a UV–Vis spectrophotometer (Agilent, Cary Eclipse, USA) at a wavelength of 204 nm. The calibration curve for Met was linear from 2 to 20 µg/mL with a correlation coefficient of R^2^ = 0.9997. The amount of 3BP was quantified using a high-performance liquid chromatography (HPLC, Agilent 1200, USA) equipped with C18 chromatographic column (150 mm × 4.6 mm, 5 μm). The mobile phase was trifluoroacetic acid aqueous solution and trifluoroacetic acid acetonitrile solution (9:1, V/V). The samples run at a flow rate of 1 mL/min. The detection wavelength was 233 nm. The calibration curve for 3BP was linear in the range 0.09375–3 mg/mL with a correlation coefficient of R^2^ = 0.9997.

The formulas for drug loading (DL) and encapsulation efficiency (EE) are as follows:

$$\text{DL}\;(\%)=\text{weight}\;\text{of}\;\text{encapsulated}\text{drug}/\text{total}\;\text{weight}\text{of}\;\text{liposome}\times 100\%;$$

$$\text{EE}\;(\%)\:=\:\text{Encapsulated}\;\text{drug}\;\text{weight}/\text{total}\;\text{drug}\;\text{weight}\times100\%.$$

### In vitro drug release

In vitro drug release was determined by a dialysis method in PBS at pH value of 6.8 with constant stirring. Briefly, nanoparticle solutions were sealed into a dialysis bag (MWCO, 3500), immersed in a beaker with PBS as release media, and stirred at 100 rpm at 37 °C. The release medium was replaced at predetermined time intervals and the released drug content was measured using a UV–visible spectrophotometer or a HPLC as described previously.

### Cytotoxicity assay

Cytotoxicity of drugs was quantitatively analyzed using the standard 3-[4,5-dimethylthiazol-2-yl]-2,5-diphenyltetrazolium bromide (MTT) assay. Briefly, 4T1 cells were seeded in 96-well cell culture plates at 8000 cells per well. When the cells reach 60–70% confluence, complete cell culture medium containing Met (0, 2.5, 5, 10, 20, 40, 60, and 80 mM) or 3BP (0, 5, 10, 20, 40, 60, 80 and 100 µM) were added and the cells were treated for 24 h. Furthermore, to examine the combination index (CI) of the two drugs, Met and 3BP, we treated 4T1 cells with different concentration ratios of Met:3BP (1000, 500, 250, 125, 83.333, and 62.5) and incubated them for 24 h. After 24 h, cytotoxicity was assessed using the MTT assay. After completing the above experiments, the optimal drug binding ratio was selected to prepare Met-3BP-Lip and Met-3BP-Lip@M1, and the cytotoxicity of the formulations was evaluated by MTT method. Briefly, 20 µL of MTT solution (5 mg/mL) was added into each well and the cells were incubated at 37 °C for 4 h. The medium was then replaced with 150 µL DMSO and mixed for 15 min on a shaker. Finally, the optical density (OD) value at the wavelength of 570 nm was measured by a microplate reader (Bio-Tek Instruments Inc., USA). Cytotoxicity calculation formula: cell viability (%) = (OD treatment group − OD blank group/OD control group − OD blank group) × 100%. CI values for drugs were calculated using Calcusyn software (version 1.0, CompuSyn, Inc., USA).

### In vitro cellular uptake

We firstly seeded 4T1 cells at a density of 1 × 10^5^ cells per well in 35 mm glass bottom dishes. When the cell density reached about 60% confluence, Cyanine5 (Cy5) fluorescently labeled NPs (Liposome or Lip@M1) were added and the cells were incubated for 2 h. After being washed 3 times with PBS (pH 7.4), the cells were fixed with 4% paraformaldehyde for 15 min at room temperature, and then washed 2 times with PBS. Finally, the nuclei were stained with 2 µg/mL DAPI for 10 min and washed 3 times with PBS before adding anti-fluorescence quenching mounting solution. Fluorescence images were obtained using a confocal laser scanning microscope (CLSM, Leica, Germany).

To further quantitatively analyze cellular uptake, the treated 4T1 cells were collected and fixed with 4% paraformaldehyde for 10 min and washed 3 times with PBS. Intracellular fluorescence intensity was measured using a flow cytometer (FACS Calibur; BD Biosciences, UK) and analyzed with FlowJo V10 software.

### Cell cycle analysis

Cell cycle assessment was performed using propidium iodide (PI) staining. Briefly, after being treated with PBS, Met, 3BP, Met&3BP, Met-3BP-Lip or Met-3BP-Lip@M1 for 24 h, the 4T1 cells were harvested, washed, resuspended in 100 µL of cold PBS, and fixed with 1 ml of pre-chilled 70% ethanol at 4 °C for 30 min. The cells were washed twice with PBS, treated with RNase A (100 µg/mL) for 30 min, and incubated with propidium iodide (50 µg/mL) for 30 min at room temperature in the dark before the analysis by flow cytometry.

### Apoptosis assay

Apoptosis assay was carried out using FITC Annexin V Apoptosis Detection Kit I (BD Biosciences, US). Briefly, the treated 4T1 cells were collected, washed twice with PBS, resuspended in binding buffer, and incubated with FITC Annexin V solution and PI solution at room temperature for 15 min in the dark before the analysis by flow cytometry.

### Glucose uptake and lactate production

Glucose and lactate concentrations were measured using a glucose concentration assay kit and a lactate assay kit (Solarbio Life Science, China) according to the manufacturer’s instructions. The treated 4T1 cells were analyzed by UV–Vis spectrophotometry.

### 3D tumor spheroid

4T1 tumor spheroids were formed in microwell devices using out established method [15, 22, 23]. When 4T1 tumor spheroids reached 200 μm in diameter, they were treated with Liposome, Lip@M1, Met, 3BP, Met&3BP, Met-3BP-Lip, or Met-3BP-Lip@M1 and new medium were added every two days. The growth of tumor spheroids was observed and recorded using a light microscope (OLYMPUS, Japan). ImageJ software was used for analysis, and the experimental results were expressed as volume change rate and circularity of tumor spheroids. Tumor spheroid volume V = (π × d_max_ × d_min_)/6. Roundness (%) = 100 − (R − r)/R × 100 (R: the radius of the smallest circumscribed circle; r: the largest inscribed concentricity degree circle).

### In vivo antitumor efficacy study

All animal experiments were approved by the Animal Ethics Committee of Nantong University. BALB/C female mice (6–8 weeks old) were purchased from the Experimental Animal Center of Nantong University. A 4T1 tumor-bearing BALB/C model was established by subcutaneously injecting 4T1 cells (1 × 10^6^ cells/mL) into the flank regions of the mice. When the tumor volume grew to 100 mm [3], all mice were randomly divided into 8 groups (n = 5) and treated with PBS, Liposome, Lip@M1, Met, 3BP, Met & 3BP, Met-3BP-Lip, and Met-3BP-Lip@M1 every 2 days via tail vein injection. The body weight and tumor volume of the mice were recorded after the injection. The tumor volume was calculated by the following formula: V = 0.5a × b^2^, where a and b represent the long and short diameters of the tumor, respectively. At the 15th day post-injection, the mice were sacrificed, and their tumors and major organs (the heart, liver, spleen, lung and kidney) were harvested and fixed in 4% paraformaldehyde solution for histology analysis.

### Histological analysis

The preserved tumors and major organs were embedded in paraffin, cut into 5 μm thick tissue sections, and stained with hematoxylin-eosin (H&E) staining for pathological evaluation. The tumor sections were also stained using Terminal deoxynucleotidyl transferase dUTP nick-end labeling (TUNEL) diaminobenzidine (DAB) apoptosis detection kit for the apoptosis analysis.

### Statistical analysis

All experiments were repeated at least 3 times, and the results were expressed as mean ± SD. All statistical analyses were performed using GraphPad Prism 6 software. Quantitative data were analyzed using a t-test (for two groups) or one-way ANOVA (for multiple groups). A p-value < 0.05 was considered statistically significant. *p value < 0.05, **p value < 0.01, ***p value < 0.001, ****p value < 0.0001.

## Results and discussion

### Preparation, characterization, and drug loading of Met-3BP-Lip@M1

**Fig. 1:**
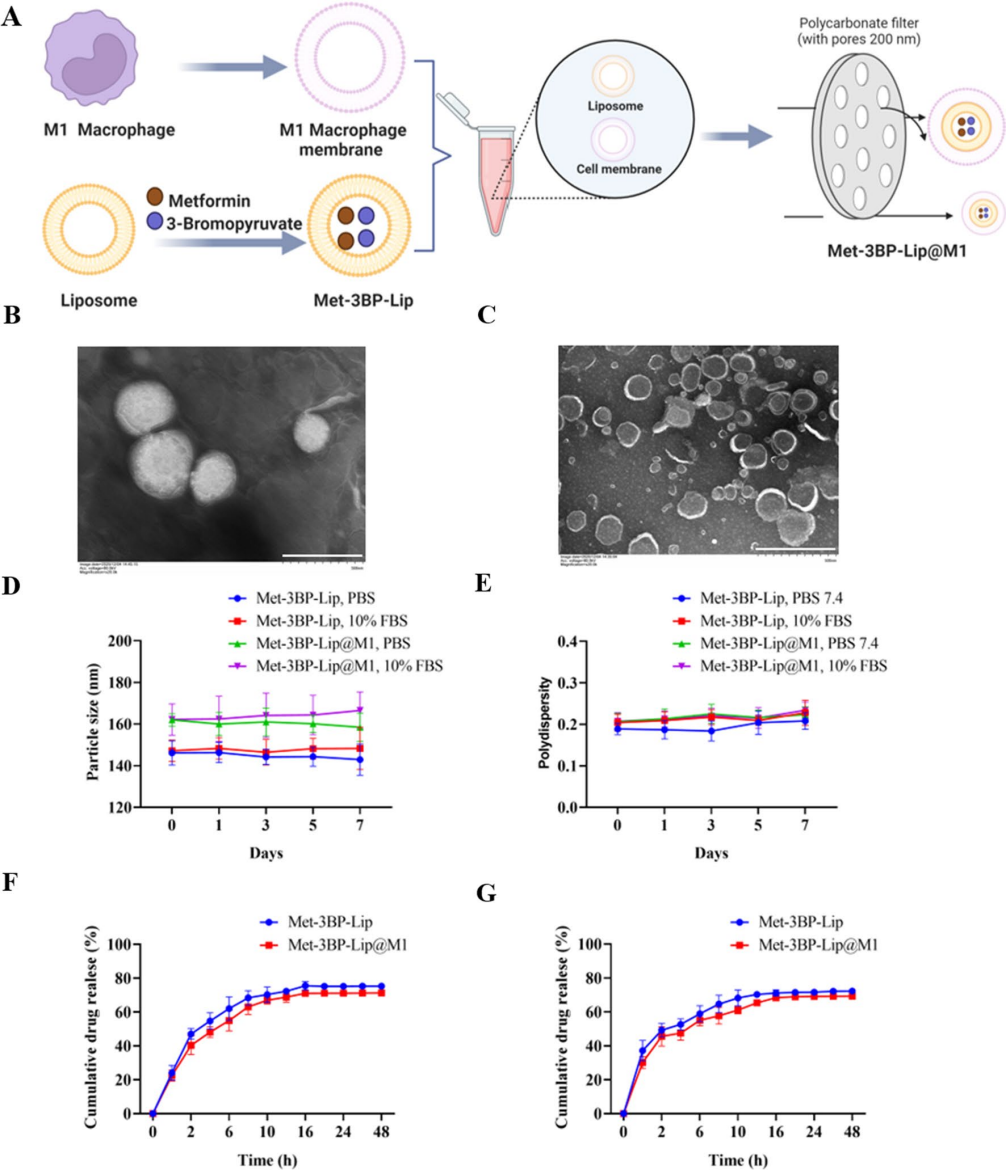
Biomimetic nanoparticle fabrication and characterizations. **A** Schematic illustration of the Met-3BP-Lip@M1 preparation process. Morphology of Met-3BP-Lip (**B**) and Met-3BP-Lip@M1 (**C**) observed by TEM. The scale bar is 500 nm. Stability evaluation of Met-3BP-Lip@M1 by measuring the changes of **D** particle size and **E** polydispersity in PBS or 10%FBS. **F** In vitro release of Met from Met-3BP-Lip and Met-3BP-Lip@M1 at pH 7.4. **G** In vitro release of 3BP. The experimental results represent the mean ± SD (n = 3)

The preparation of the M1 membrane-coated nanomedicine with drug loading is illustrated in Fig. 1A. Briefly, liposomes encapsulating Met and 3BP (Met-3BP-Lip) were synthesized using thin-film hydration method. M1 macrophage membrane and Met-3BP-Lip were co-extruded using a liposome extruder to form biomimetic Met-3BP-Lip@M1. As shown in Fig. 1B, C, the morphology of Met-3BP-Lip and Met-3BP-Lip@M1 was measured by TEM, which showed uniform spherical shape. Also, Stability of Met-3BP-Lip and Met-3BP-Lip@M1 was also investigated over 7 days. Neither the particle size nor the polydispersity remarkably changed when the nanoparticles were incubated in PBS or 10%FBS at pH 7.4 (Fig. 1D, E). In addition, the hydrodynamic size, surface charge and loading efficiency of Met-3BP-Lip and Met-3BP-Lip@M1 were determined and summarized in Table 1. The measurements revealed that Met-3BP-Lip had a size of 147.77 ± 5.78 nm and a polydispersity index (PDI) of around 0.23 ± 0.02, while the size of Met-3BP-Lip@M1 increased to 162.19 ± 4.29 nm with a PDI of around 0.21 ± 0.02 due to M1 macrophage membrane coating. Zeta potential of Met-3BP-Lip@M1 decreased to − 31.65 ± 1.28 mV compared to Met-3BP-Lip, which has a zeta potential of − 22.72 ± 2.12. The increased particle size as well as decreased zeta potential preliminarily indicated that cell membrane-functionalized biomimetic liposomes was successfully prepared. The drug loading and encapsulation efficiency of Met and 3BP were detected by a UV–Vis spectrophotometer at 204 nm and high-performance liquid chromatography at 233 nm, respectively. According to the detection results, the drug loading was 13.20 ± 2.07% and 0.22 ± 0.014% for Met and 3BP, respectively, while the encapsulation efficiency was 69.21 ± 3.89% and 71.74 ± 4.13% for Met and 3BP, respectively. In addition, the drug release profile of Met-3BP-Lip@M1 was recorded at 37 °C and pH 7.4 over 48 h. The results showed that sustained release of Met and 3BP were achieved by the formulations, reaching 69.21 ± 3.89% and 71.74 ± 4.13%, respectively (Fig. 1F, G).

**Table 1:** Physical properties and drug loading efficiency of the nanoparticles

Sample	Hydrodynamic size D_h_^a^ (nm)	Polydispersity (PDI)	Zeta-potential (mV)	Drug loading efficiency (%)		Entrapment efficiency (%)	
Liposome	120.41 ± 2.29	0.24 ± 0.01	− 27.92 ± 1.91				
Lip@M1	133.56 ± 1.94	0.21 ± 0.03	− 32.77 ± 2.35				
Met-3BP-Lip	147.77 ± 5.78	0.23 ± 0.02	− 22.72 ± 2.12	Met	13.65 ± 2.64%	Met	71.46 ± 3.37%
				3BP	0.23 ± 0.021%	3BP	75.23 ± 4.67%
Met-3BP-Lip@M1	162.19 ± 4.29	0.21 ± 0.02	− 31.65 ± 1.28	Met	13.20 ± 2.07%	Met	69.21 ± 3.89%
				3BP	0.22 ± 0.014%	3BP	71.74 ± 4.13%

^a^Determined by DLS

### Cellular uptake

**Fig. 2:**
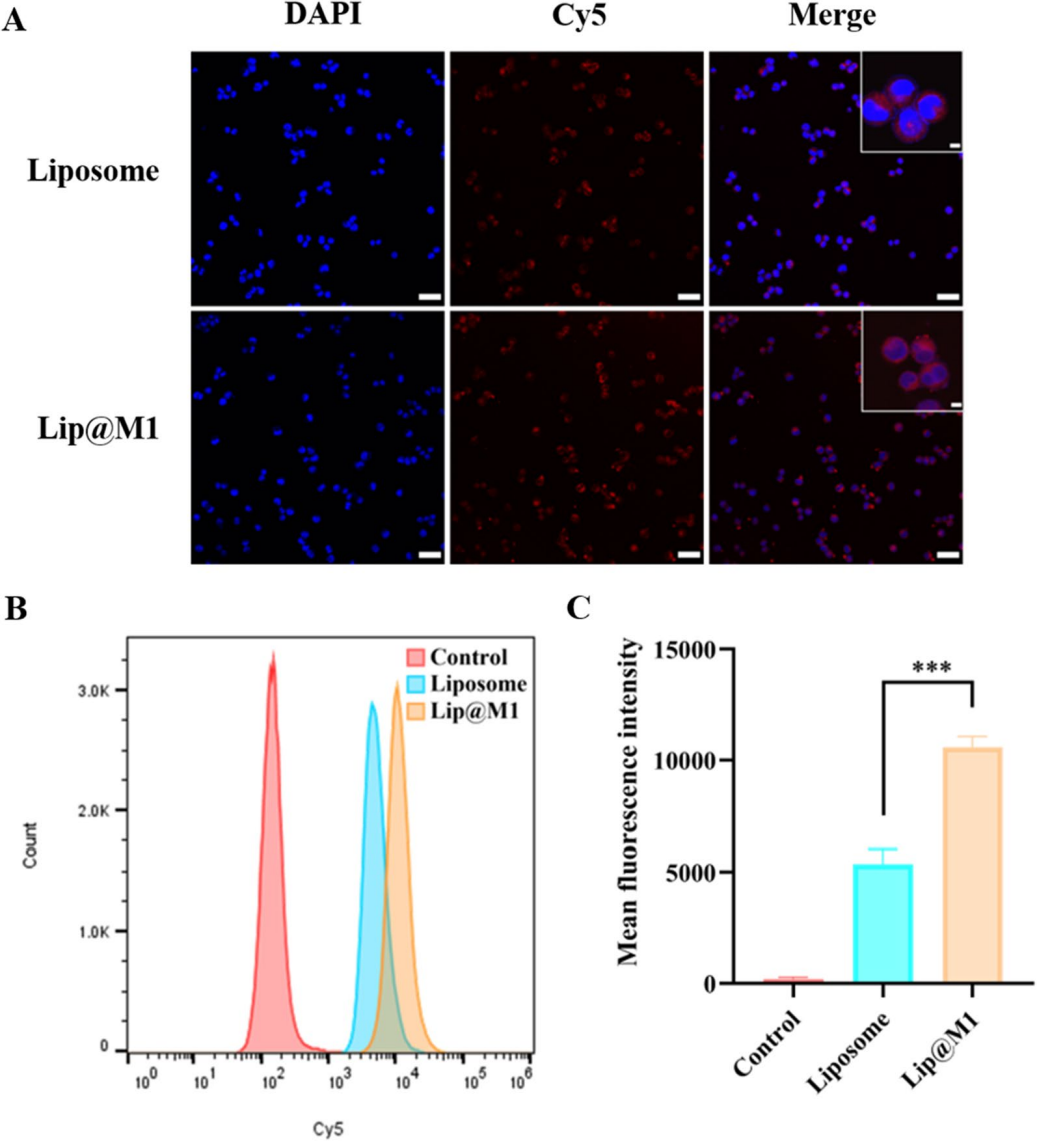
Cellular uptake. **A** Subcellular distribution of Liposome and Lip@M1 on 4T1 breast cancer cells was observed by a confocal microscopy. The scale bar is 50 μm (5 μm for the enlarged images). **B** Flow cytometry analysis of nanoparticle uptake by 4T1 cells after 2 h treatment. **C** Quantitative analysis of intracellular Cy5 mean fluorescence intensity. The experimental results represent the mean ± SD (n = 3)

To verify whether macrophage membrane-functionalized liposomes could enhance the in vitro uptake ability of 4T1 cells, we used Cy5 fluorescent dye-labelednanoparticles with or without M1 cell membrane (Liposome or Lip@M1) and co-incubated them with 4T1 cells for 2 h. The cellular uptake was then examined using a confocal microscopy. The results showed that the intracellular fluorescence intensity of the Lip@M1 group was significantly higher than that of the Liposome group (Fig. 2A). This was also confirmed by the quantitative analysis of flow cytometry. The intracellular fluorescence intensity of the Lip@M1 group was 1.97 times greater than that of the Liposome group at 2 h. The above results indicate that the functionalization of the M1 macrophage membrane can endow Lip@M1 with a stronger uptake ability. This M1 macrophage membrane-enabled cellular uptake is likely closely related to the high expression of integrins in macrophage membranes [24].

### In vitro evaluation of the anticancer activity of Met-3BP-Lip@M1

**Fig. 3:**
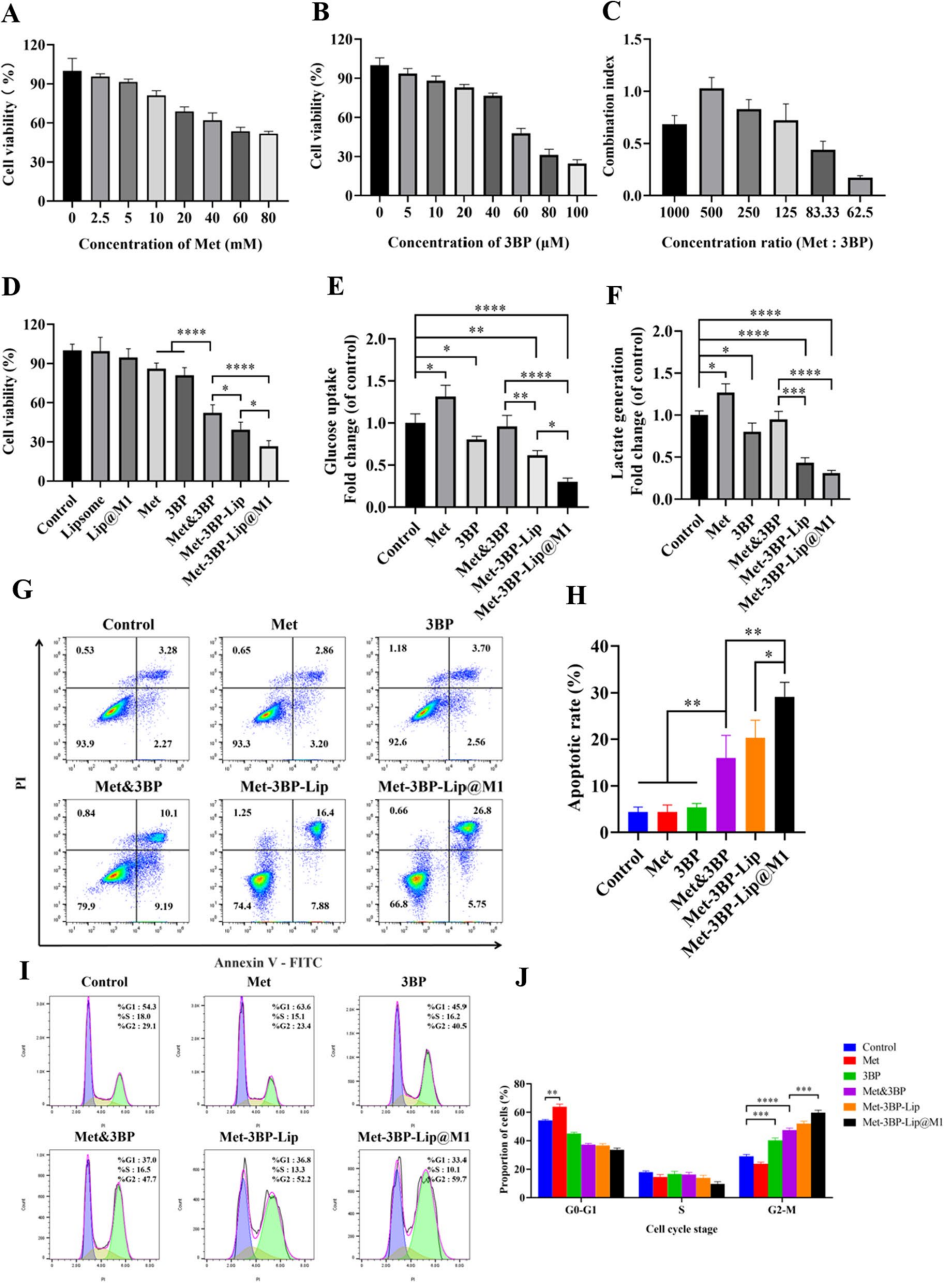
The cytotoxicity and mechanism of action studies. **A** Cytotoxicity of different concentrations of Met towards 4T1 cells. **B** Cytotoxicity of different concentrations of 3BP towards 4T1 cells. **C** Combined index (CI) of different concentration ratios of Met and3BP. **D** Cytotoxicity of different drug formulations towards 4T1 cells. **E** The effects of the drug formulations on glucose consumption. **F** The effects of the drug formulations on lactate generation. **G** Flow cytometry analysis histogram of cell cycle distribution after treatment. **H** Quantitative analysis of cell cycle phase distributions for each group. **I** Flow cytometry analysis of apoptosis after treatment. **J** Percentage of apoptotic and necrotic cell populations in the treated 4T1 cells. The experimental results represent the mean ± SD

In order to achieve the best cancer inhibitory effect, we investigated the synergistic cytostatic effect of Met and 3BP on 4T1 cells after 24 h of co-incubation. The anticancer effects of Met and 3BP were analyzed using Calcusyn software. Both Met and 3BP exhibited dose-dependent cytotoxicity with IC50 values of 68 mM and 55 µM, respectively (Fig. 3A, B). Synergistic effect was quantified by calculated the combination index (CI) using the Chou–Talalay method (Fig. 3C) [22]. The synergistic effect was greatest when the drug molar ratio (Met:3BP) was 62.5. The cytotoxicity of Met-3BP-Lip@M1 NPs was carried out using MTT assay. Liposome and Lip@M1 had no significant cytotoxic effect on 4T1 cells (Fig. 3D). Both free 3BP and Met had slightly anti-cancer activities against 4T1 cells compared to the control group. Combined drug treatment (Met&3BP) reduced cellular survival rates to 52.19 ± 6.17%, indicating that combination of Met and 3BP inhibited cell growth to some extent. In addition, compared with the Met&3BP group and Met-3BP-Lip group, the Met-3BP-Lip@M1 group had a better curative effect. This may be attributed to the improved cellular uptake as well as immunostimulatory properties of M1 macrophage membrane as also demonstrated in our previous studies [15, 16].

### Glucose consumption and lactate production analysis

Cancer cells normally have boosted glucose uptake and lactate production to maintain their high proliferation rates. It has been reported that Met, an inhibitor of mitochondrial complex I, can reduce tumorigenesis by causing bioenergetic stress in cancer cells [25]. 3BP is also used for anticancer therapy because of inhibiting glycolytic enzymes and ATP production. To investigate the effects of biomimetic nanomedicines in energy exhaustion, we measured glucose levels and lactate production in the culture medium used to grow 4T1 cells after drug treatment (Fig. 3E, F). The glucose consumption and lactate production of Met-treated 4T1 cells were approximately 1.31-fold and 1.27-fold higher, respectively, than those of the control group. This is because metformin inhibited complex 1 of the electron transport chain in mitochondria, which made cancer cells reliant on glycolysis for ATP synthesis. Our results showed that 3BP alone reduced the glucose and lactate levels to certain extend. On the contrary, there were no statistically significant differences in glucose uptake and lactate production in the Met&3BP group in comparison to the control group. More importantly, compared with the Met&3BP group, our prepared Met-3BP-Lip and Met-3BP-Lip@M1 had more significant inhibitory effects on glucose consumption and lactate production. Our data demonstrated that Met-3BP-Lip@M1 had a better capacity to synergistically limit energy supply in cancer cells via dual regulation on energy metabolism. The decoration of the M1 macrophage membrane, which facilitated drug targeting and cellular uptake, also contributed to this metabolism reprogramming.

### Cell cycle and apoptosis studies

Annexin V/PI staining was performed to further evaluate biomimetic nanomedicine-induced apoptosis in 4T1 cells. As indicated in Fig. 3G, H, compared with the control, 4T1 cells exposed to Met or 3BP did not cause obvious apoptosis after 24 h of treatment, which is consistent with the findings of the cell viability analysis. Whereas the Met&3BP group significantly increased the apoptotic rate of 4T1 cells to 15.98 ± 4.88%. It is worth noting that the apoptotic rates in the Met-3BP-Lip and Met-3BP-Lip@M1 groups were higher, reaching 20.31 ± 3.83% and 29.16 ± 3.09%, respectively, and the majority of them were in a late apoptosis state. Particularly, the total apoptosis rate of Met-3BP-Lip@M1 was approximately 1.82-fold more effective than that of Met&3BP. The increased apoptotic potential brought on by Met-3BP-Lip@M1 may be associated with a better internalization of the macrophage membrane as a coating. According to the results, Met&3BP had a synergistic effect to induce strong metabolic stress and inhibit cell activity, leading to apoptosis. Met-3BP-Lip@M1 group induced the highest apoptosis rate, suggesting limiting energy metabolism in cancer cells using the biomimetic drug formulation resulted in marked apoptotic impact.

The cell cycle assay was then performed using propidium iodide (PI) staining and analyzed by a flow cytometer (Fig. 3I, J). Compared with the control group, free Met significantly caused cell cycle arrest in the G1 phase in 4T1 breast cancer cells, while free 3BP dramatically increased the number of cells in the G2 phase, from 29.10 ± 1.30% to 40.30 ± 1.60%, resulting in G2 phase cell cycle arrest. Met&3BP group had a considerable increase in the number of cells in the G2 phase, suggesting the induction of G2 phase cell cycle arrest. Notably, Met-3BP-Lip and Met-3BP-Lip@M1 increased the number of cells in the G2 phase to 52.00 ± 1.64% and 59.70 ± 1.80%, respectively, which further enhanced cell cycle arrest in the G2 phase. These results indicate that these biomimetic nanocarrier-based co-delivery system encapsulated with Met and 3BP synergistically promoted cell cycle arrest, which contributed to cancer cell death for anti-cancer treatment.

### Tumor spheroid inhibition study

**Fig. 4:**
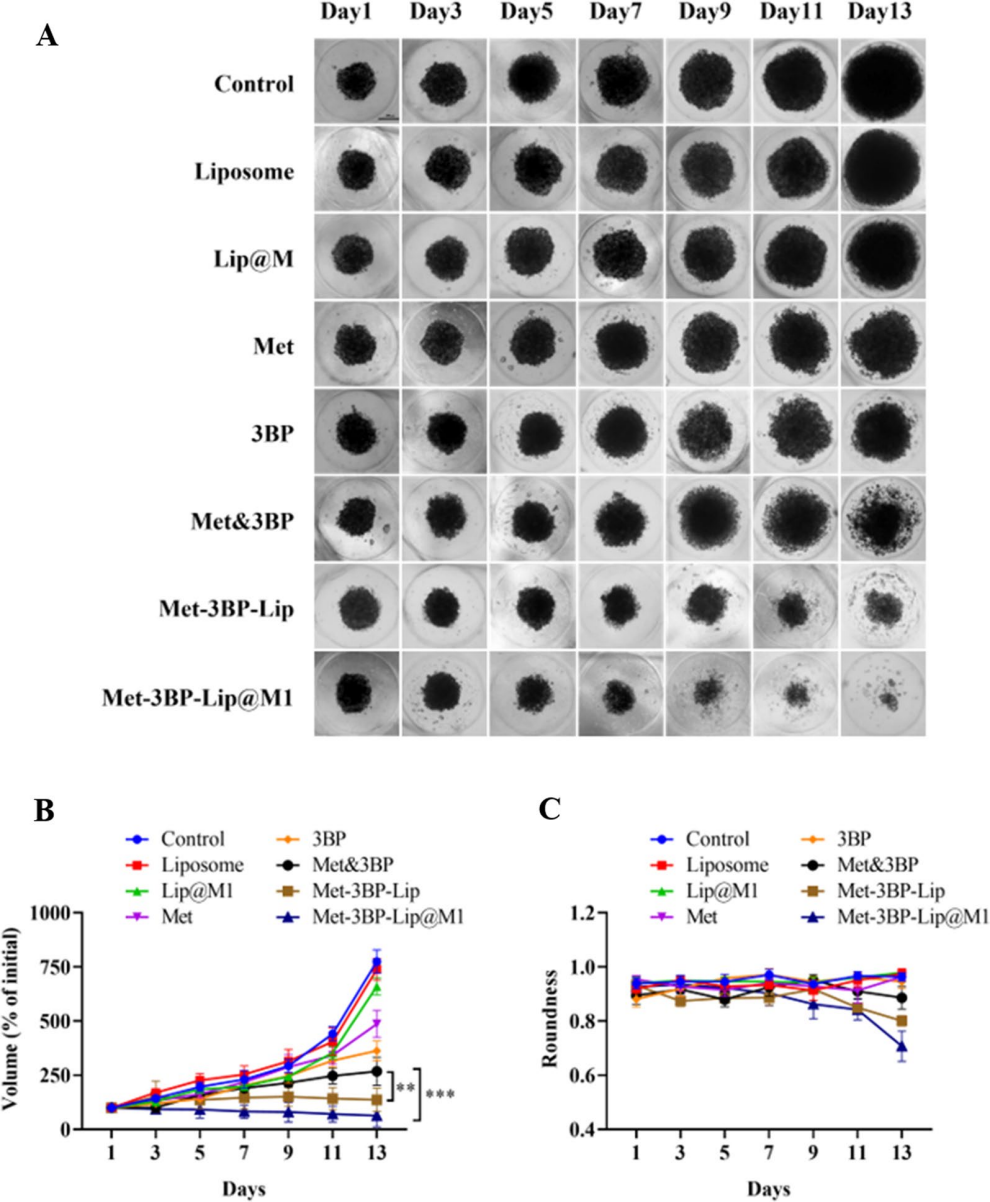
**A** Morphological observation of the 4T1 tumor spheroids treated with different drug formulations. Scale bar = 200 μm. **B** The relative volume change curve of 4T1 tumor spheroids after drug treatment. **C** Circularity curve of 4T1 tumor spheroids after drug treatment

In vitro 3D tumor spheroids are widely used three-dimensional cell culture models for anticancer drug screening and evaluation due to their similarity to the physiological environment of tumor tissue. We successfully constructed 4T1 tumor spheroids in vitro and treated them with different drug formulations. The Fig. 4A showed the morphology of the tumor spheroids in each group on day 13 after treatment. Compared with the control group, free Met and 3BP slightly reduced the growth of 4T1 tumor spheroids, while the treated 3D spheroid maintained the aggregate-like structure without further breakdown. We observed that Met&3BP, Met-3BP-Lip, and Met-3BP-Lip@M1 were able to progressively damage the extracellular matrix of the treated spheroids, leading to reduced cell adhesion and enhanced cell death. Moreover, Met-3BP-Lip@M1 had the strongest inhibitory effect on the growth of 4T1 tumor spheroids, and the tumor spheroids in this group gradually shrank in size over time (Fig. 6B). In addition, we discovered that the tumor spheroids in the Met-3BP-Lip@M1 treatment group gradually fragmented at the periphery with an irregular shape by comparing the spheroid roundness results (Fig. 6C). It has been demonstrated that Met-3BP-Lip@M1 can effectively penetrate tumor spheroids, demolish the aggregate-like structure of tumor spheroids, and inhibit the formation and growth of tumor spheroids, possibly due to the boosted cellular uptake and cell death mechanisms.

**Fig. 5:**
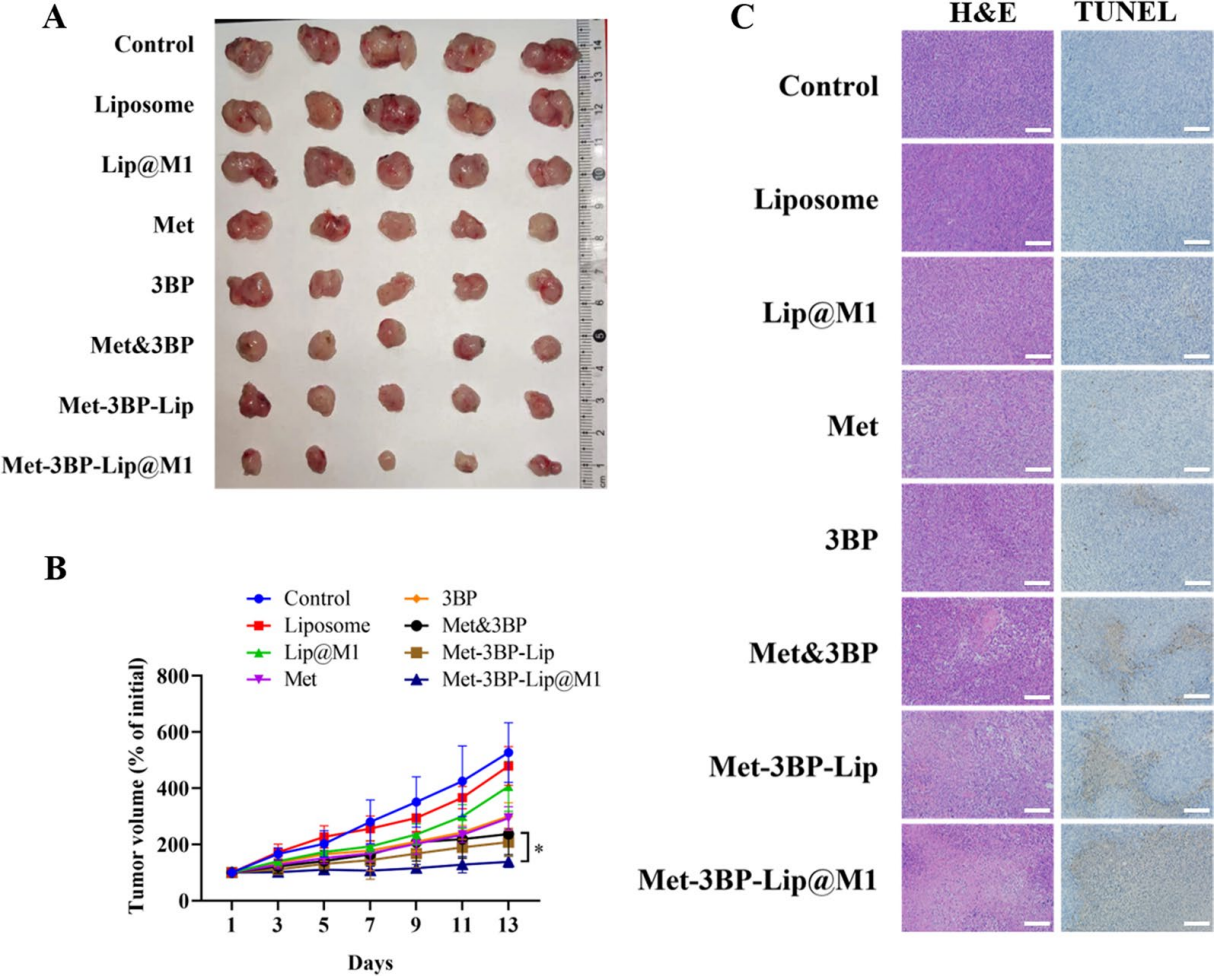
**A** Photographs of the excised tumors obtained from different groups at the end of the treatments (n = 5). **B** Relative tumor volume change curve after different treatments. **C** Representative histological images of 4T1 tumor slices after different treatments using H&E staining (scale bar = 100 μm) and TUNEL staining (scale bar = 100 μm)

### In vivo therapeutic efficacy and safety assessment of the starvation therapy

**Fig. 6:**
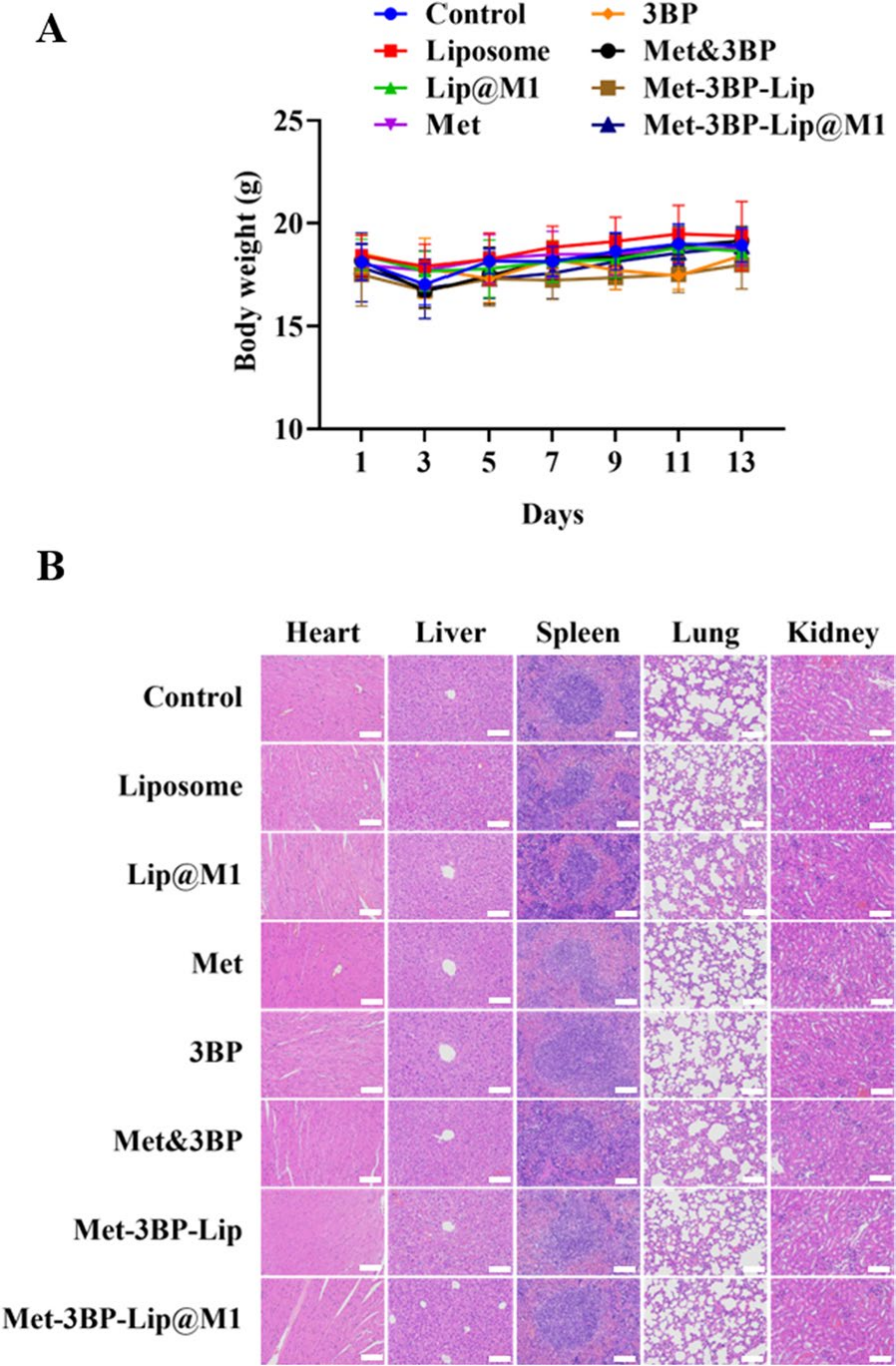
In vivo biosafety assessment. **A** Body weight of the mice injected with different treatments. **B** Representative histological images of H&E-stained slices of the major organs. Scale bar = 100 μm

To evaluate the in vivo anti-tumour efficiency of Met-3BP-Lip@M1, the treatment was administered into 4T1-bearing BALB/c mice through the tail vein every other day after the tumor establishment. Tumor growth was monitored, recorded and analyzed to compare the therapeutic efficacy (Fig. 5A, B). The data demonstrated that Met, 3BP, and Met&3BP groups considerably slowed down the tumor growth compared with the control and nanocarriers without drug loading. Met-3BP-Lip@M1 possessed supreme tumor suppression efficiency in comparison with other groups. This improved therapeutic efficacy is consistent with in vitro experiments. In addition, the histological analysis of the tumor tissue using H&E staining showed that Met-3BP-Lip@M1 substantially reduced the tumor cell number, which verified the excellent synergistic starvation therapeutic effects of (Fig. 5C). We also observed that Met-3BP-Lip@M1 induced the highest levels of apoptosis using TUNEL staining among all the groups, which is consistent with the results obtained by in vitro apoptosis assay. The excellent anti-tumor effects of Met-3BP-Lip@M1 suggest that the engineered biomimetic nanoplatform could significantly benefit starvation therapy for breast cancer. No noticeable weight loss was observed during the treatment period (Fig. 6A). Furthermore, the histological analysis of the major organs displayed negligible tissue abnormality of the treatment groups (Fig. 6B), suggesting the biocompatibility of the formulations for in vivo therapeutic applications. Collectively, these findings demonstrated that the energy-limiting effects of Met-3BP-Lip@M1 were specific for tumor tissues.

## Conclusion

We designed and developed a biomimetic therapeutic nanoplatform based on M1 macrophage membrane functionalization and quantitative loading of two energy metabolism inhibitors. The engineered Met-3BP-Lip@M1 showed targeted uptake and cancer-killing effects in 4T1 breast cancer cells. In addition, by examination of the cell death mechanisms, we found that Met-3BP-Lip@M1 synergistically promoted cell cycle arrest and apoptosis of cancer cells, which is closely associated with the decrease of oxidative phosphorylation and glycolysis. Our in vivo studies demonstrated that Met-3BP-Lip@M1 efficiently eliminated tumor growth, while enhanced local tumor cell apoptosis in the tumor region. Our biomimetic nanoformulation exhibited superior therapeutic efficacy and biosafety for cancer treatment. This novel starvation therapy against breast cancer provides a promising strategy for clinical applications.

## Data Availability

The data used and analyzed during this study are included in this article and are available from the first authors on reasonable request.
